# STAT activation status differentiates leukemogenic from non-leukemogenic stem cells in AML and is suppressed by arsenic in t(6;9)-positive AML

**DOI:** 10.18632/genesandcancer.39

**Published:** 2014-11

**Authors:** Claudia Oancea, Brigitte Rüster, Boris Brill, Jessica Roos, Maria Heinssmann, Gesine Bug, Afsar Ali Mian, Nathalie Andrea Guillen, Steven M. Kornblau, Reinhard Henschler, Martin Ruthardt

**Affiliations:** ^1^ Department of Hematology, Goethe University, Frankfurt, Germany; ^2^ Department of Transfusion Medicine and Immunohematology, Goethe University, Frankfurt, Germany; ^3^ Georg-Speyer-Haus, Institute for Biomedical Research, Frankfurt am Main, Germany; ^4^ Section of Molecular Hematology and Therapy, Department of Leukemia, The University of Texas, MD Anderson Cancer Center, Houston, TX, USA; ^5^ University Clinic Munich, Department for Transfusion Medicine, Cell Therapy and Hemostaseology, Munich, Germany

**Keywords:** leukemia initiating cell, t(6:9), STAT5, arsenic trioxide

## Abstract

Acute myeloid leukemia (AML) is characterized by an aberrant self-renewal of hematopoietic stem cells (HSC) and a block in differentiation. The major therapeutic challenge is the characterization of the leukemic stem cell as a target for the eradication of the disease. Until now the biology of AML-associated fusion proteins (AAFPs), such as the t(15;17)-PML/RARα, t(8;21)-RUNX1/RUNX1T1 and t(6;9)-DEK/NUP214, all able to induce AML in mice, was investigated in different models and genetic backgrounds, not directly comparable to each other. To avoid the bias of different techniques and models we expressed these three AML-inducing oncogenes in an identical genetic background and compared their influence on the HSC compartment *in vitro* and *in vivo*.

These AAFPs exerted differential effects on HSCs and PML/RARα, similar to DEK/NUP214, induced a leukemic phenotype from a small subpopulation of HSCs with a surface marker pattern of long-term HSC and characterized by activated STAT3 and 5. In contrast the established AML occurred from mature populations in the bone marrow. The activation of STAT5 by PML/RARα and DEK/NUP214 was confirmed in t(15;17)(PML/RARα) and t(6;9)(DEK/NUP214)-positive patients as compared to normal CD34+ cells. The activation of STAT5 was reduced upon the exposure to Arsenic which was accompanied by apoptosis in both PML/RARα- and DEK/NUP214-positive leukemic cells. These findings indicate that in AML the activation of STATs plays a decisive role in the biology of the leukemic stem cell. Furthermore we establish exposure to arsenic as a novel concept for the treatment of this high risk t(6;9)-positive AML.

## INTRODUCTION

Acute myeloid leukemia (AML) is maintained by an accelerated proliferation of blasts and the aberrant stem cell capacity of poorly defined leukemic stem cells (LSC) along with a block in differentiation [1]. In order to develop more efficient therapy options in AML a great effort is placed upon targeting LSCs with the aim to eradicate the disease [2, 3]. AMLs are frequently associated with specific chromosomal translocations and their related aberrant fusion proteins (AAFPs)[4] such as PML/RARα, DEK/NUP214 or RUNX1/RUNX1T1. Expression of AAFPs in murine hematopoietic stem and progenitor cells (HSPC) recapitulates many features of the leukemic phenotype such as the differentiation block and increased self renewal [5-9].

Differences between the various mouse models of AML are inherent to the technical and biological characteristics of a given model and appear to be related to the type of cells that are targeted for transformation by the AAFPs.

PML/RARα-positive leukemia models are based on a transgenic approach driven by regulatory elements of cathepsin G or hMPR8, or a knock-in [10-13], as well as on transduction/transplantation models [5, 9, 14]. A transduction/transplantation model targets the expression of PML/RARα to either lin^−^ progenitors or Sca1^+^/lin^−^ [5, 15].

Despite detectable effects of RUNX1/RUNX1T1, such as increased replating efficiency, expansion of the HSPC compartment and impairment in myeloid differentiation, transgenic mouse models gave a RUNX1/RUNX1T1-related leukemic phenotype only after treatment with mutagenic agents or in specific genetically modified backgrounds that separately provided a “second hit” [16-18]. The knock-in model of RUNX1/RUNX1T1 under the control the Sca1 promoter induced a myeloproliferative disease, suggesting that targeting the “correct” stem/progenitor cell is important for the transformational potential of RUNX1/RUNX1T1 [19]. Only a truncated form and the “short” RUNX1/RUNX1T1ex9 isoform allowed an efficient leukemia induction in a transduction/transplantation model [8, 20].

For the DEK/NUP214, the AAFP of the high risk t(6;9)-positive AML, no transgenic model is actually available. In a transduction/transplantation model DEK/NUP214 targets a primitive HSC population for leukemic transformation, probably not targetable in transgenic models [6].

STATs are frequently constitutively activated in leukemia [21, 22] and considered to play an important role for the LSC in mouse models of AML [23, 24]. Over-expression of a constitutively active STAT5 has been shown to increase self-renewal of human CD34+ HSCs [25, 26]. The loss of STAT5 impairs LT-HSC maintenance both in human normal and leukemic hematopoiesis [27]. Activation of STATs is not restricted to tyrosine phosphorylation, but they may be also phosphorylated at serine residues situated in the C-terminal transactivation domain (TAD) [28]. The contribution of serine phosphorylation to the transcriptional activity of tyrosine phosphorylated STAT proteins is controversial and might be STAT-protein specific but also promoter and/or cell context specific [28]. In chronic lymphocytic leukemia (CLL) STAT3 is constitutively phosphorylated exclusively at serine 727 and exhibits DNA-binding and transcription activating activity on known STAT3 target genes [29]. Recently a direct relationship between AAFP and STAT activation has been shown [24, 30].

Based on a common technical platform of murine *in vitro* and *in vivo* AML-models we here asked i) whether the cell that is virtually first subjected to the chromosomal aberration or is it first transformed by an AAFP (hereafter referred to as the “leukemia initiating cell - L-IC) can be identified for each type of AAFP; ii) whether this cell type is phenotypically different from the cells that sustain leukemic growth in already established leukemia (hereafter referred to as the “leukemia maintaining cell - L-MC); iii.) whether activation of STATs plays a role in the determination of the L-IC and is pharmacologically targetable.

## RESULTS

### AAFPs induce leukemia from HSPCs with a low penetrance and a long latency

**Figure 1:**
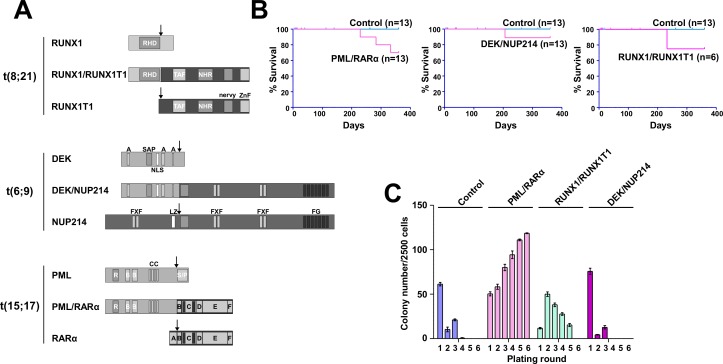
Efficiency of leukemia induction and the replating capacity of murine HSPCs expressing the AAFPs (A) Modular organization of the fusion proteins and the translocation partners in t(15;17), t(8;21), and t(6;9). PML/RARα - PML: R - Ring domain; B - B-boxes: CC - coiled coil oligomerization interface. RARα: A - AF-1 transactivation domain; C - DNA binding domain; D - nuclear corepressor complex binding domain; E - nuclear localization signal; ligand binding domain, AF-2 transactivation domain, RXR interaction domain. RUNX1/RUNX1T1 - RUNX1: RHD - runt homology domain. RUNX1T1: TAF - TAF homology domain; HHR - hydrophobic heptad repeat; nervy - nervy homology domain; ZnF - zinc finger domain. DEK/NUP214 - DEK: A - acidic regions; SAP - scaffold attachment factor; NLS - nuclear localization signal. NUP214: FXF - repeat motifs; LZ - leucine zipper; FG - repeat motifs. Arrows: breakpoints in leukemia. (B) Sca1^+^/lin^−^ BM cells were infected with retroviruses as described in reference [6]. At 5 hours post-infection, 5×10^4^ cells/mouse were transplanted into sublethally irradiated mice to determine their leukemogenic potential. The survival curves show the frequency of ill mice that succumbed to disease within one year. The number of mice for each group is indicated. (C) Sca1^+^/lin^−^ BM cells were retrovirally infected and plated in semi-solid medium to determine the serial replating potential. The colony number was counted on days 8–10. The cells were then harvested and serially replated. We show one representative experiment (+/−SD) of at least three performed experiments that were each conducted in triplicate.

To define the L-IC in AML, we compared the leukemogenic potential of three different AAFPs. We chose the AAFP of two good-risk AMLs — the t(8;21)-related RUNX1/RUNX1T1 and the t(15;17)-related PML/RARα — and of one poor risk AML — the t(6;9)-related DEK/NUP214 (Figure 1A). Retrovirally transduced Sca1^+^/lin^−^ HSPCs (5×10^4^) were inoculated into sublethally irradiated recipient mice. As shown in Figure 1B, DEK/NUP214 and RUNX1/RUNX1T1 both induced leukemia with a low efficiency and long latency. PML/RARα, induced an AML with signs of differentiation in the BM and without signs of differentiation in the spleen. In contrast to the DEK/NUP214-induced AML without signs of differentiation, RUNX1/RUNX1T1 caused an AML with signs of differentiation according to the Bethesda classification [31] (Supplementary Figure S1).

In summary, all AAFPs induced leukemia from the immature Sca1^+^/lin^−^ HSPC compartment with a low efficiency and long latency.

### Differential effects of AAFPs on the replating potential of ST- and LT-HSC and progenitor populations

PML/RARα or RUNX1/RUNX1T1 increase the replating efficiency of HSPCs [6, 9], which is considered to be related to their effects on differentiation, proliferation and self-renewal potential of these progenitors [3, 13]. Here we directly compared the effects of these AAFPs on the *in vitro* replating efficiency of HSPCs [6, 9, 14]. DEK/NUP214 did not increase the replating efficiency [6]. In contrast to PML/RARα, which conferred immortality, as shown by its capacity to allow at least 10 replatings (data not shown), RUNX1/RUNX1T1-positive HSPCs became exhausted after the 5th plating (Figure 1C)

**Figure 2:**
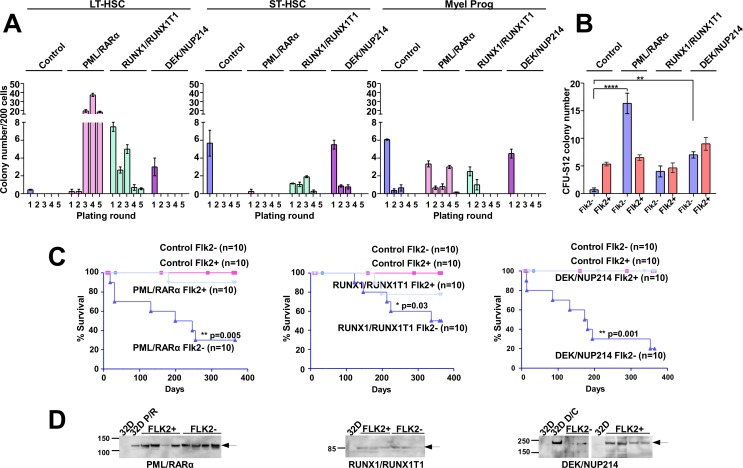
The effect of the AAFPs on the replating efficiency, CFU-S12 potential and the leukemogenic potential of ST- and LT-HSC (A) Sca1^+^/lin^−^ BM cells were infected with retroviruses as indicated in reference [6]. At 12 hours post-infection, the cells were sorted for LT-HSCs (Sca1^+^/c-Kit^+^/lin^−^/Flk2^−^), ST-HSCs (Sca1^+^/c-Kit^+^/lin^−^/Flk2^+^) and myeloid progenitors (MPs) (Sca1^−^/c-Kit^+^/lin^−^). The sorted cells were then plated in semi-solid medium supplemented with mIL-3, mIL6 and mSCF to determine the serial replating potential. In the first plating for LT-HSC 300 cells/well, for ST-HSC 500 cells/well and for MP 3×10^3^ cells/well were seeded. The colonies were counted after 8–10 days, prior to each replating round, and the cell number was assessed for further replatings. For the following plating rounds 2.5×10^3^ cells/well were seeded. For platings with cell numbers lower than 2.5×10^3^ cells/well, all cells were replated. In order to keep comparable the different samples the colony counts are reported to 200 cells seeded. One representative experiment (+/−SD) is shown. Three experiments were performed in total, with similar results, and each experiment was performed in triplicate. (B) Differential effects of the AAFPs on the potential of LT- und ST-HSCs to form colonies in a CFU-S12. The cells harvested from the first plating in the semi-solid medium were inoculated into lethally irradiated recipient mice for the CFU-S12 assay. The animals were sacrificed at day 12, and the spleen colonies were counted. We show one representative experiment of at least three performed experiments (all had similar results). Each group was comprised of 3 mice. (C) Differential effects of the AAFPs on the potential of LT- und ST-HSCs to induce leukemia. The survival curves show the frequency of recipients that succumbed to disease after receiving the cells harvested from the CFU-S12 spleens. Each group contained 10 mice. (D) Expression of the AAFPs in leukemic mice inoculated with the Sca1^+^/c-Kit^+^/lin^−^/Flk2^−^ (Flk2-) and in healthy mice inoculated with the Sca1^+^/c-Kit^+^/lin^−^/Flk2^+^ (Flk2+) populations, respectively, as determined by Western blot. Mock-transduced 32D cells or cells that stably expressed the respective AAFPs were used as controls.

As it remains unclear to which extent an increased replating efficiency is related to aberrant self-renewal, we investigated the effects of the AAFPs on the replating efficiency of long term (LT-), short term (ST-) hematopoietic stem cells (HSC) and progenitors [1]. GFP-positive Sca1^+^/lin^−^ cells expressing PML/RARα, RUNX1/RUNX1T1 or DEK/NUP214 were sorted for ST- (Sca1^+^/c-Kit^+^/lin^−^/Flk2^+^) and LT-HSC (Sca1^+^/c-Kit^+^/lin^−^/Flk2^−^) and myeloid progenitors (Sca1^−^/c-Kit^+^/lin^−^)(MP) as previously reported [6]. Despite the fact that only viable cells but no colonies were visible in the first two plating rounds, PML/RARα-positive LT-HSCs efficiently initiated colony-formation starting from the third plating, and it was not exhausted even after 6 platings (Figure 2A). In contrast colony formation by RUNX1/RUNX1T1-positive LT-HSC was already exhausted after four platings, and DEK/NUP214-positive LT-HSCs did not induce colonies after the first plating (Figure 2A). A slightly increased replating efficiency was observed for the RUNX1/RUNX1T1- and DEK/NUP214-, but surprisingly not for the PML/RARα-positive ST-HSCs. The replating capacity of PML/RARα-positive MPs exhausted after five platings (Figure 2A). Control and DEK/NUP214-transduced MPs did not contain any colony forming cells beyond the second plating (Figure 2A).

These findings indicate that the immortalization of PML/RARα-positive HSPCs is based on the transformation of cells with a LT-HSC phenotype in contrast to RUNX1/RUNX1T1-positive HSPCs whose serial replating capacity rapidly exhaust from both subpopulations [9].

### The AAFPs increase the CFU-S12-forming capacity of LT-HSC

To determine the effects of the AAFPs on the self-renewal potential of ST-HSCs and LT-HSCs, we performed a CFU-S12 assay on cells derived from the first plating round in semi-solid medium (Figure 2A). CFU-S12 detects early progenitors and ST-HSC in normal hematopoiesis [32]. In our assays empty vector-infected ST-HSCs (Control) produced a higher number of spleen colonies than Control LT-HSCs, providing a control for the functional integrity of the cells comparable to normal hematopoiesis (Figure 2B). PML/RARα significantly increased colonies from the LT- but not from the ST-HSC compartment. RUNX1/RUNX1T1 slightly increased the CFU-S12 capacity from the LT-HSCs but not from the ST-HSC population, whereas DEK/NUP214 increased the CFU-S12 potential from both compartments (Figure 2B). The hematopoietic origin of the spleen colonies was confirmed on HE-stained spleen sections (data not shown).

These data suggest that the aberrant colony formation in semi-solid medium recapitulates the aberrant stem cell capacity of PML/RARα, but not of the other AAFPs.

### PML/RARα and DEK/NUP214 induce leukemia from the LT-HSC, whereas RUNX1/RUNX1T1 induces leukemia from both cell types with low efficiency and long latency

Recently, we showed that DEK/NUP214 initiates leukemia from LT-HSCs [6]. In order to compare the different AAFPs, the CFU-S12 spleens were homogenized and 2×10^5^ cells were inoculated into sublethally irradiated recipient mice as already described [6]. As shown in Figure 2C, the cells originating from the PML/RARα-, RUNX1/RUNX1T1- or DEK/NUP214-positive LT-HSCs induced leukemia with a high penetrance of 70%, 50% or 80%, respectively. In contrast, the cells originating from the ST-HSCs induced leukemia with a penetrance of 10%, 20% or 0% respectively. None of the healthy mice showed signs of leukemia or pre-leukemia, as indicated by their normal white blood cell counts (WBCs) and normal spleen and liver sizes upon euthanasia (data not shown and Supplementary Figure S2). To ensure that the differences in leukemia induction were not due either to a lack of transgene expression or to engraftment failure, we performed Western blots of BM and spleen from mice inoculated with cells of LT- and ST-HSC origin, leukemic (at diagnosis) and healthy (at the end of the investigation time - 12 months), respectively. Both ill and healthy mice inoculated with either LT- or ST-subpopulations fully expressed the respective transgenes, indicating the differential transformation potential of the AAFPs in LT-HSCs compared with ST-HSCs (Figure 2D).

### PML/RARα-positive leukemia is maintained by committed progenitors

**Figure 3:**
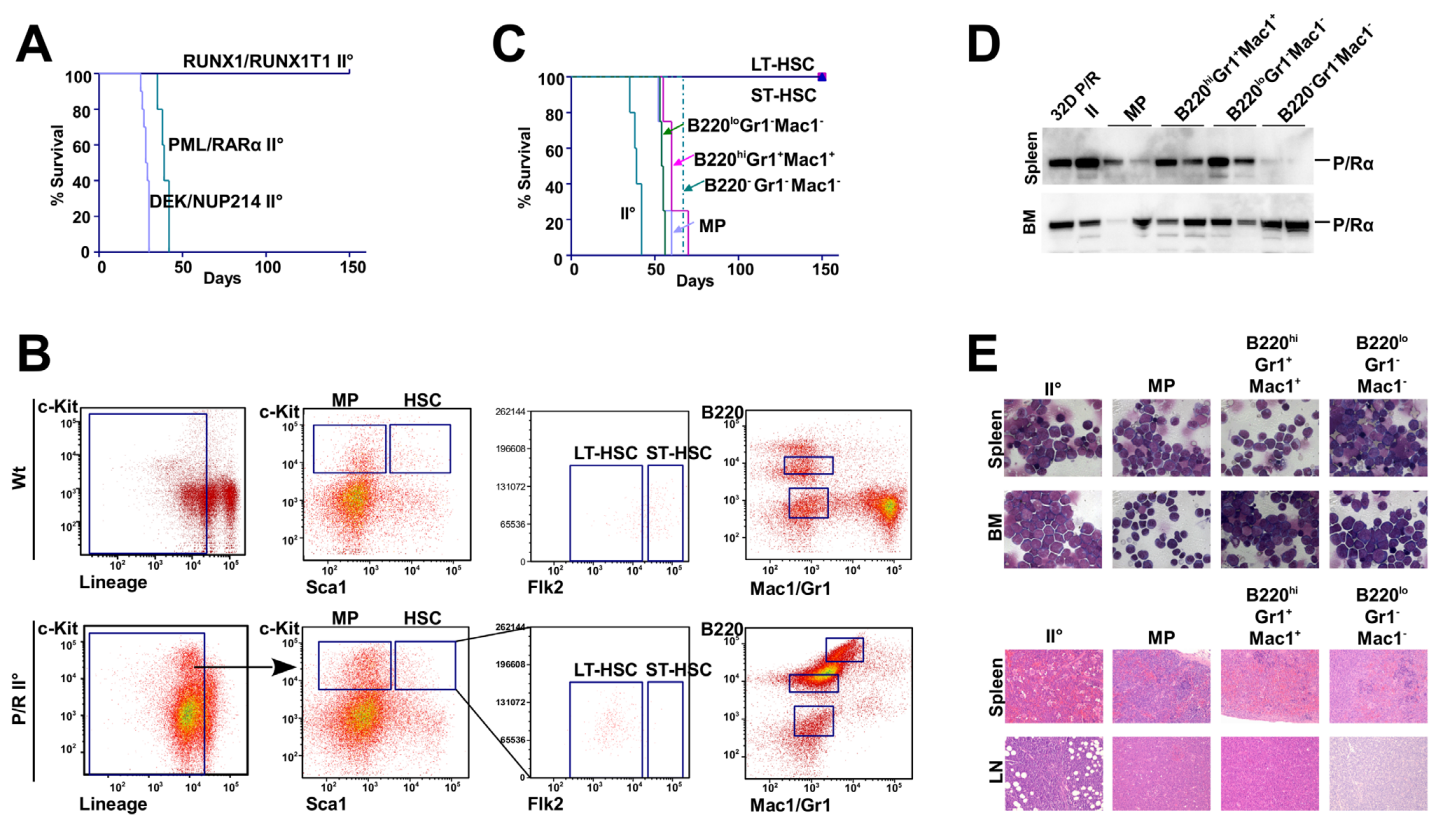
The secondary transplantation of AAFP-induced leukemia and the leukemia maintaining subpopulation (A) For the induction of secondary leukemia, frozen samples from primary leukemic mice were inoculated into sublethally irradiated mice. Frozen spleen cells isolated from primary leukemia induced by the indicated AAFPs and derived from LT-HSC, as described previously, were inoculated into sublethally irradiated recipient mice. The survival curves show the recipients succumbing to disease after receiving 2×10^4^ cells/mouse. (B) Fresh spleen cells from secondary leukemic mice were sorted based on the expression of c-Kit, Sca1, Flk2 and the lineage markers (MP, LT- and ST-HSC) or of B220 and the myeloid markers Mac1 and Gr1. The sorted cell populations were then inoculated into sublethally irradiated recipient mice to determine the leukemogenic potential of the sorted subpopulations. The sorting protocol is shown for the indicated subpopulations within the secondary PML/RARα-positive leukemia (C) The survival curves of recipients succumbing to disease after receiving the sorted subpopulations. (D) Expression of PML/RARα in mice harboring secondary (II) leukemia and leukemia from the MP and the indicated subpopulations, as determined by Western blot. (E) Morphological and histological analysis of samples from secondary leukemia or leukemia from the indicated subpopulations.

The “leukemia maintaining” cell population of an already established DEK/NUP214-positive leukemia is not restricted to the original L-IC population [6]. To identify the cell population that maintains the PML/RARα- or RUNX1/RUNX1T1-positive leukemias, we transferred 2×10^4^ cells from leukemic mice, with a tumor load of at least 80%, into sublethally irradiated secondary recipient mice. Mice inoculated with PML/RARα- or DEK/NUP214-positive leukemic cells developed leukemia with a latency of approximately 4 weeks and a penetrance of 100%. In contrast, RUNX1/RUNX1T1- positive leukemic cells did not re-establish leukemia in secondary recipients (Figure 3A). To compare the cell population maintaining PML/RARα-positive leukemia with the L-IC population, we sorted the principal subpopulations for transplantation into sublethally irradiated recipients (Figure 3B). The established PML/RARα-positive leukemia was composed of 73% lin^lo/-^ cells, 65% of which were already committed Sca1^−^/c-Kit^−^ cells, 13.5% were Sca1^−^/c-Kit^+^ and 1.5% presented the stem-cell phenotype of Sca1^+^/c-Kit^+^. Because L-ICs were found within the Sca1^+^/c-Kit^+^/lin^−^ population, we sorted for Sca1^−^/c-Kit^+^/lin^lo^ (MPs), and for Flk2^+^ ST-HSCs and Flk2^−^ LT-HSCs within the Sca1^+^/c-Kit^+^/lin^−^ cell compartment. Based on findings that in some mouse leukemia models L-ICs were found in the B220^+^ population, and in human AMLs a lymphoid-primed multipotent population with leukemia maintaining capacity exists [2, 33], we sorted for Gr1, Mac1 and B220 expression (Figure 3B). We inoculated 5×10^3^ cells from each population into recipient mice. With the exception of ST- and LT-HSCs, all populations gave rise to a highly aggressive immature leukemia within 80 days (Figure 3C). The almost identical latency to leukemia onset strongly suggested that a similar number of cells sustained leukemia development. All populations generated an identical phenotype compared to the primary leukemia with AML (Figure 3E). The population with a LT- and ST-HSC phenotype did not induce leukemia within one year (Figure 3C). This is noteworthy because it clearly represented the population that was transformed by PML/RARα to initiate leukemia. The presence of the transgene was confirmed by Western blot (Figure 3D).

In summary, these data show that the AAFPs PML/RARα and DEK/NUP214 initiate leukemia by transforming a small subpopulation of LT-HSCs, but both maintain already established leukemia from already committed cell populations in the BM suggesting a difference between L-ICs and L-MCs.

### PML/RARα- and DEK/NUP214-positive leukemic cells exhibit enhanced STAT signaling

**Figure 4:**
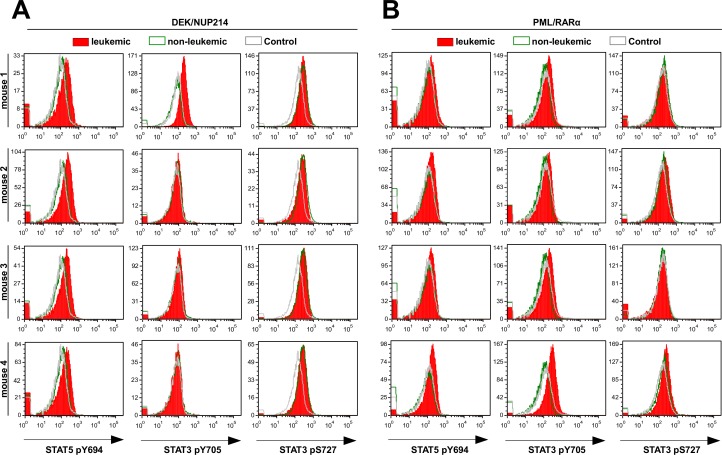
Activation of STAT proteins in leukemic versus non-leukemic AAFP-positive spleen cells Activation status of STAT5 (Y694) and STAT3 (Y705 and S727) was analyzed by flow cytometry on freshly thawed leukemic (Flk2-) and non-leukemic (Flk2+) DEK/NUP214-positive (A) and PML/RAR-positive (B) cells (3 × 10^5^ cells per FACS tube) compared to control cells from mice inoculated with empty vector transduced Flk2^−^ cells (Control Flk2-). Histograms show four leukemic mice for each AAFP group. Similarly, 4 non-leukemic and 4 control mice were analyzed. Data from one representative mouse from the later groups is shown.

Given the fact that the differences in leukemia induction between the LT- and ST-HSCs are related to differences in the cell populations targeted by the AAFPs, we examined whether the STAT signaling is differentially activated in leukemic versus non-leukemic AAFP-positive cells in the absence of any cytokine stimulation. STAT activation was addressed by intracellular FACS staining for STAT3pY705, STAT3pS727 and STAT5pY694. We observed a slight activation of STAT3 at pY705 in both PML/RARα- and DEK/NUP214-positive leukemic cells as compared to the PML/RARα- and DEK/NUP214-positive non-leukemic and control cells (Figure 4A,B). DEK/NUP214 itself seemed to induce a high STAT3pS727 in both leukemic and non-leukemic cells compared to controls. PML/RARα-positive leukemic and non leukemic cells showed very low or absent STAT3pS727 levels as compared to controls. In contrast, STAT5 was clearly activated at Y694 in leukemic DEK/NUP214- and to a lower extent, in leukemic PML/RARα-positive cells as compared to non-leukemic cells or controls (Figure 4A,B).

### STAT activation is accompanied by a constitutively activated JAK2 in DEK/NUP214-positive leukemic cells and down regulation of CD45 in leukemic cells expressing DEK/NUP214 and PML/RARα

**Figure 5:**
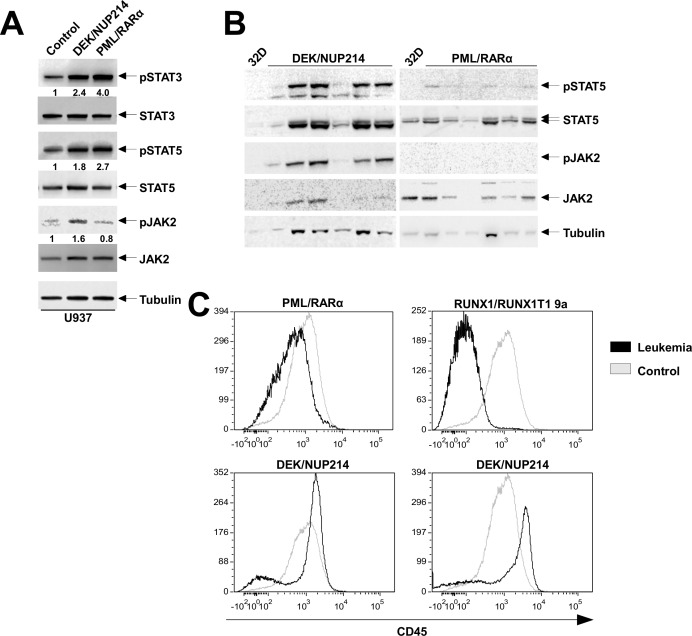
Activation of JAK/STAT signaling is accompanied by the down regulation of CD45 (A) Analysis of STAT3, STAT5, and JAK2 activation by western blotting in DEK/NUP214- and PML/RARα-positive U937 cells. Numbers below represent the fold change of the phosphoprotein intensity normalized to the corresponding total level and compared to the control lane (B) Western blot analysis of STAT5 and JAK2 and their phosphorylated form (Y694 and Y1007/1008, respectively) in DEK/NUP214-positive and PML/RARα-positive leukemic cells (C) Flow cytometric analysis of CD45 for bone marrow cells from PML/RARα, DEK/NUP214 and RUNX1/RUNX1T1 leukemia mice compared to bone marrow from wild-type mice.

To disclose whether the STAT activation is related to the expression of the AAFPs, we studied the activation status of STAT3 and 5 in the U937 cell line stably expressing PML/RARα or DEK/NUP214. Serum- and cytokine-starved 32D cells served as negative controls for the phospho-proteins. We found that both STAT3 and STAT5 were activated to a much higher extent in the presence of AAFPs as compared to control cells (Figure 5A).

To determine whether the increased STAT phosphorylation is due to activated upstream signals, we investigated the activation status of JAK2. The activation of JAK2 was addressed by phosphorylation on Y1007/1008. We detected an increased activation of JAK2 in DEK/NUP214-, but not in PML/RARα-positive U937 cells (Figure 5A) or in the respective leukemic cells (Figure 5B).

STAT activation can also be regulated by the phosphatase activity of CD45 as shown for RUNX1/RUNX1T1-positive leukemia [30]. Here we show that CD45 was down regulated in the PML/RARα-positive leukemic cells, similar to RUNX1/RUNX1T1-positive cells, whereas DEK/NUP214 down regulated CD45 expression only in a fraction of the leukemic cell population (Figure 5C).

Taken together, these data show that the leukemic phenotype induced by PML/RARα or DEK/NUP214 is associated with a constitutive activation of STAT3 and/or 5 and that the targeted expression of the AAFPs to different subpopulations in the BM might have different intracellular signaling responses that contribute to leukemia development. Notably, the STAT activation appears to be induced by activated JAK2 only in DEK/NUP214- but not in PML/RARα-positive leukemic cells, where it might be due to the loss of CD45 expression.

### STATs are activated in t(6;9)-positive AML samples

**Figure 6:**
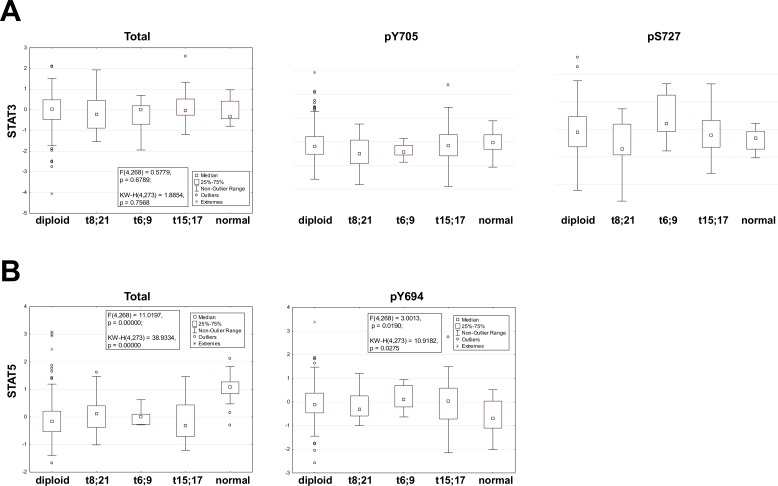
STAT3 and 5 activation in t(6;9), t(15;17) and t(8;21)-positive AML samples Box plots showing the expression range of Stat3 (total, pY705 and pS727) and Stat5 (total and pY694) stratified by cytogenetic group: Diploid, t(8;21), t(6;9), t(15;17) and normal bone marrow derived CD34+ cells (Normal) are presented. The box shows the 25-75% range with the median shown by the central square (□), outlier by a circle (o) and extremes by an asterisk (*). Statistical results for the F-test and Kruskal-Wallis including the degrees of freedom and the associated p-value are listed.

To confirm these data we investigated the level of STAT3 or STAT5 activation in primary patient samples. The activation status of STATs was assessed by RPPA in a cohort of 531 newly diagnosed, previously untreated, AML patients that included patients with t(6;9) (n=6), t(15;17) (n=20) and t(8;21) (n=15). The RPPA allowed to determine the expression levels of total STAT1, 3 and 5 and the phosphorylation status of STAT1 (Y701), STAT3 (Y705 and S727), STAT5 (Y694) and STAT6 (Y641). STAT1 and 6 served as specificity control. Details on methodology and patient cohort are reported by Kornblau [34, 35]. The level of expression was compared both to patients with diploid cytogenetics comprising FLT3- ITD-positive as well as negative samples, and to bulks of CD34^+^ samples from healthy donors. The cohort of t(6;9) patients included 4 of 6 cases positive for FLT3- ITD. Notably there was no difference in the level of total or phospho-STAT between diploid FLT3-WT or FLT3- ITD cases for any of these (data not shown). The levels of total or phospho-STAT1 or phospho-STAT6 were similar between the diploid control group and the three AAFP groups. Phospho-STAT1 was significantly decreased in all leukemia groups as compared to normal CD34^+^ samples (Supplementary Figure S3). Phospho-STAT6 was significantly increased in all leukemia groups in comparison to the normal CD34^+^ samples (Supplementary Figure S3). Levels of total STAT3 did not differ among the leukemia groups and in comparison to normal CD34^+^ samples. Regarding the activation of STAT3 no differences were revealed for STAT3pY705 between all groups but a significantly higher level of STAT3pS727 was seen only among patients with t(6;9)(Figure 6A). Total STAT5 was significantly lower in all leukemia groups with respect to the CD34^+^ group. STAT5pY694 was higher in all leukemia groups as compared to the CD34^+^ groups (Figure 6B).

This data supports the murine model findings that STAT3 and STAT5 are phosphorylated in t(6;9) AML and that this is independent of FLT3-ITD. Levels of phospho-STAT3 or 5 were not higher in either t(15;17) or t(8;21) cases compared to the diploid cases (regardless of FLT3 status) (Figure 6A,B). In a separate RPPA that compared expression in bulk, CD34^+^ and CD34^+^/CD38^−^ populations derived from primary AML samples the level of expression of total STAT3 and phospho-STAT1, 5 and 6 were all significantly higher (P <0.0004) in the CD34^+^/CD38^−^ compartment compared to the other populations, supporting the concept that STAT activation is relevant to leukemic stem cell biology [36].

### Similar to PML/RARα, DEK/NUP214-positive leukemia responds to ATO treatment and DEK/NUP214 mediates arsenic trioxide (ATO) induced apoptosis

**Figure 7:**
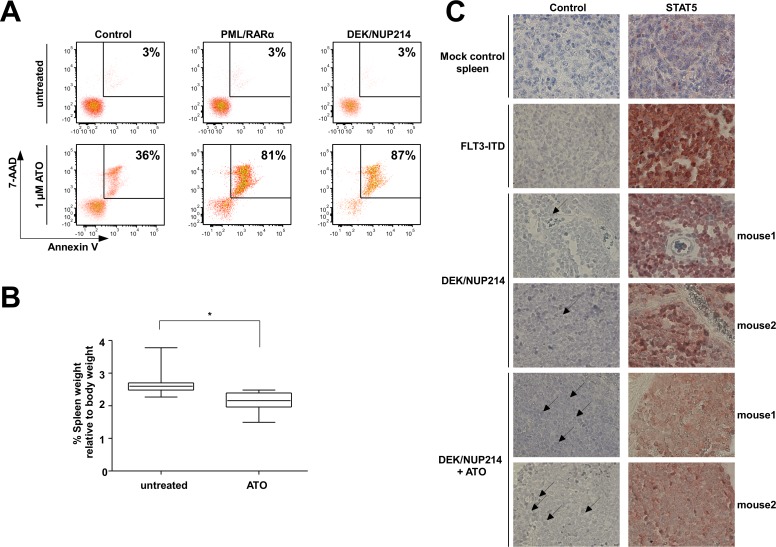
Responsiveness of DEK/NUP214-positive cells to ATO treatment (A) 7-AAD and Annexin V staining of mock-control, DEK/NUP214- and PML/RARα-positive U937 cells after treatment with 1 μM ATO for five days. Percentage of dead cells (7-AAD and Annexin V positive) is shown. (B) Frozen spleen cells isolated from DEK/NUP214-leukemic mice were inoculated into sublethally irradiated recipient mice. Five days after transplantation the mice from the treatment group received ATO for 14 days. The control group received PBS. Mice were sacrificed and spleen size was measured at the first signs of illness among the control group (n=8 mice/group) (C) Immunohistochemical staining of formalin-fixed paraffin-embedded spleen sections from mice described in 7B to determine the nuclear localization of STAT5 in untreated *vs.* ATO-treated group. As controls, spleen sections from mice injected with mock-transduced HSPCs or from mice with FLT3-ITD driven leukemia were used as negative and positive control, respectively.

Activated STATs have been reported to be efficiently targeted by ATO [37]. ATO targets the leukemic stem cells and induces a high rate of long-term relapse free survival in PML/RARα-positive APL patients [15, 38, 39, 40]. We investigated whether activated STATs may mediate response to ATO treatment not only in PML/RARα but also in DEK/NUP214-positive leukemia. The expression of the STAT5 activating factors, such as PML/RARα, RUNX1/RUNX1T1 and BCR/ABL, sensitize U937 cells to ATO-induced apoptosis [41]. Thus we exposed U937 cells stably expressing DEK/NUP214 cells to ATO. Apoptosis was measured at day 5 by 7-AAD/Annexin V staining. As controls we used empty vector and PML/RARα-expressing U937 cells. The expression of DEK/NUP214 sensitized the U937 cells to ATO induced apoptosis to a similar extent as PML/RARα (Figure 7A).

To further explore the response of DEK/NUP214-positive leukemia to ATO we exposed mice inoculated with DEK/NUP214-positive leukemic cells to ATO for 14 days starting at day 5 after inoculation. The mice were sacrificed at the first signs of disease in the untreated group. Disease degree was assessed by spleen size. The mice treated with ATO had a significantly reduced spleen size as compared to controls (Figure 7B) accompanied by a reduction of nuclear STAT5 upon ATO treatment compared to untreated mice as determined by immunohistochemistry and immunofluorescence of spleen sections (Figure 7C and Supplementary Figure S4). In addition, multiple fragmented nuclei were observed in the treated group (black arrows in the control panel in Figure 7C) as a sign of ongoing apoptosis (see also Supplementary Figure S5).

These data indicate that the STAT activation mediates the responsiveness of DEK/NUP214-positive leukemia cells towards ATO-induced apoptosis.

## DISCUSSION

The aim of our study was to define the L-IC of different AML subentities induced by specific chromosomal abnormalities. We found that the L-IC of PML/RARα-positive leukemia is different from the L-MC, as already described for DEK/NUP214-positive leukemia [6]. The L-MC for RUNX1/RUNX1T1 could not be defined because the related leukemia was not retransplantable, indicating that RUNX1/RUNX1T1 does not completely transform HSPCs.

Our experimental approach was purely qualitative in order to answer the question of whether we can enrich the very rare cell populations which are transformed by the AAFPs from the LT- or ST-HSC, respectively. We hypothesized that such an enrichment may occur in two ways: either by proliferation of the transformed cells themselves or by the outgrowth of other cell populations with a stem cell immunophenotype, still able of serial replating or colony formation in CFU-S12, but not able anymore to finally give origin to leukemia.

The AAFPs confer different characteristics to the HSPCs, as shown by their effects on the serial replating potential. An increased serial replating efficiency is caused by a combination of increased proliferation and block of differentiation. Here we prove that the increase of serial replating potential really accounts for the aberrant self-renewal capacity of PML/RARα-positive HSPCs. In fact, the PML/RARα-positive cells can be replated more than 10 times (data not shown). Whereas the CFCs in the first platings originate from progenitors, the following platings are supported by LT-HSCs, which acquire colony forming potential. Thus, our results suggest that the cells with LT-HSC phenotype expressing PML/RARα proliferate very well even if they do not give origin to colonies in the first platings and with the time acquire/reach the differentiation level to give CFU. For PML/RARα the differentiation block does not allow the above mentioned “outgrowth” and therefore induces the “immortalization”. We show that the population which gives rise to this “immortalization” is the subpopulation with the LT-HSC phenotype, whereas the other subpopulations exhaust their replating capacity at the latest after the 5th plating.

In contrast, the CFCs in RUNX1/RUNX1T1-positive HSPCs originate from the LT-HSCs and exhaust, suggesting that RUNX1/RUNX1T1 is unable to increase the self-renewal potential of the HSPCs. The slightly increased replating efficiency of DEK/NUP214-positive HSPCs appears to be supported by the ST-HSC compartment, without an increase in self-renewal potential, as revealed by the almost immediate exhaustion. In cells with a LT-HSC phenotype DEK/NUP214 only slows proliferation but does not block differentiation to a detectable level, and the viable cells harvested after the first plating still maintain stem cell capacity and thus are able to give rise to CFU-S12 and to induce leukemia.

Our findings prove that targeting the right cell, rather than secondary biological effects, is the decisive factor for leukemogenic transformation by the AAFPs. DEK/NUP214 and PML/RARα exert their leukemogenic potential on a very small subpopulation of HSC explaining the low penetrance of leukemia from PML/RARα- and DEK/NUP214-transduced HSPCs. Both generate a leukemic phenotype from LT-HSCs. RUNX1/RUNX1T1 initiated AML from HSPCs, but the L-IC was not clearly attributable to either an LT- or a ST-HSC subpopulation.

The L-IC can be defined as the cell in which the initial transforming event occurs, whereas L-MC refers to the cell within the tumor bulk able to maintain the leukemic growth *in vivo* [3]. Our analysis focused on the L-IC transformed by the AAFPs as a result of the primary genetic event: the chromosomal translocation. The fact that we define the LT-HSCs as L-ICs for PML/RARα-induced AML is apparently in contrast to recent findings, which locate the LSC to a committed progenitor compartment with a promyelocytic phenotype [11, 13]. In contrast to our model, these findings were obtained using an already established leukemia based on the expression of PML/RARα driven by promoters mainly active in committed progenitors [11, 13], but also in the LT-HSC compartment, even at a lower level [42]. These models define perfectly the L-MC but not the L-IC. Here we extend the findings of the transgene models for the L-MC potential of PML/RARα to a broader population that comprises not only the promyelocytes but also other subpopulations excluding immature HSC [11, 13].

Differences between DEK/NUP214- and PML/RARα-positive leukemic and healthy mice comprise activated STATs confirming recent data on RUNX1/RUNX1T1 and MOZ/TIF2 [24, 30]. There is evidence suggesting that low abundance clones with higher levels of phospho-STAT5 present at diagnosis are selected for and can become the dominant clone at relapse [43]. Our correlative data from the proteomic analysis of primary patient samples support the finding that levels of activated STATs are higher in t(6;9) samples and in stem cell enriched CD34^+^/CD38^−^ fractions.

The activation of STATs by DEK/NUP214 is accompanied by JAK2 activation, whereas PML/RARα seems to influence STATs by down-regulating CD45, as recently described also for RUNX1/RUNX1T1 [30].

The clinical significance of our data is given by the relationship between STAT-activation and response to ATO [37], known to target the LSC in PML/RARα-positive APL [15]. The response to ATO shown for DEK/NUP214-positive murine leukemia suggest a possibility of a stem cell active treatment for high risk t(6;9)-positive AML patients.

Taken together, our data define the target cells of leukemogenic transformation by the AAFPs PML/RARα, RUNX1/RUNX1T1 and DEK/NUP214. Furthermore, we provide evidence that the activated STATs may represent a good target in the leukemic stem cells, which may justify an extension of ATO-based therapy of AML.

## MATERIALS AND METHODS

### Ethics statement

All animal procedures were approved by the Regierungspräsidium Darmstadt (approval number F39/08). The human cell samples were collected at the M. D. Anderson Cancer Center on Institutional Review Board (IRB)–approved protocol Lab01-473. Informed consent was obtained in accordance with the Declaration of Helsinki. Samples were analyzed under an IRB-approved laboratory protocol (Lab05-0654).

### Plasmids

The cDNAs encoding PML/RARα, RUNX1/RUNX1T1 and DEK/NUP214, and the resulting retroviral PINCO vectors, have been described previously [6, 9]

### Isolation of Sca1^+^/lin^−^ HSPCs, retroviral infection and sorting of long term (LT)-, short term (ST)- HSCs and myeloid progenitors (MPs)

Sca1^+^/lin^−^ HSPCs were isolated from 8-12 week-old female C57BL/6N mice (Janvier, St. Berthevin, France) as already described [6]. The cells were pre-stimulated for 2 days in DMEM/10% fetal calf serum (FCS) (Hyclone, Bonn Germany), mIL-3 (20 ng/mL), mIL-6 (20 ng/mL) and mSCF (100 ng/mL) (Cell Concepts, Umkirch, Germany). Ecotropic Phoenix packaging cells were transfected with the retroviral vectors described above as described before [6]. The infection efficiency was adjusted to 70%, as assessed by detecting GFP-positive cells. For fluorescence activated cell sorting (FACS), the cells were stained with fluorochrome-conjugated antibodies against lineage markers, c-Kit, Sca1 and Flk2 (BD Biosciences, Le Pont de Claix, France), as described before [6]. Anti-CD45 staining was performed accordingly.

### Replating efficiency

At day 5 post-infection, 5×10^3^ cells/mL Sca1^+^/lin^−^ cells were plated in methyl-cellulose (MC) supplemented with mIL-3, mIL-6, and mSCF (Stem Cell Technologies, Vancouver, Canada). The number of colony-forming units (CFU) was determined on day 10 after plating. The cells were washed out for serial replating and FACS analysis. For the GFP-positive LT-HSCs (Sca1^+^/c-Kit^+^/lin^−^/Flk2^−^) 300 cells/well, ST-HSCs (Sca1^+^/c-Kit^+^/lin^−^/Flk2^+^) 500 cells/well and for MPs (Sca1^−^/c-Kit^+^/lin^−^) 3×10^3^ cells/well were plated. For the following plating rounds 2.5×10^3^ cells/well were seeded. For platings with cell numbers lower than 2.5×10^3^ cells/well, all cells were replated. In order to keep comparable the different samples the colony and the cell counts are reported to 200 cells seeded.

### Transduction/transplantation model of leukemia

Recipient female C57BL/6N mice were irradiated with 4.5 Gy. 5×10^4^ of retrovirally transduced Sca^+^/lin^−^ HSPCs per mouse were inoculated by tail vein injection. The mice were sacrificed at the first appearance of morbidity [6]. Whole BM cells and spleen cells were cytospun and stained with May-Grünwald-Giemsa stain. For surface marker analysis, Ficoll enriched mononuclear cells (MNCs) were used.

### Colony-forming unit-spleen day 12 assay (CFU-S12) and leukemic potential of CFU-S12 derived spleen cells

Cells from the first plating round were harvested and inoculated into lethally irradiated (11 Gy) recipient mice. The number of cells transplanted was 3×10^4^/mouse for sorted LT-HSCs and 2×10^5^/mouse for ST-HSCs with the exception of DEK/NUP214 where only 1×10^5^/mouse were available. At day 12 the spleens were either homogenized for further transplantations or fixed in Tellysniczky's fixative for colony counting, as described previously [15]. Spleen sections were stained with hematoxylin/eosin (HE) for morphological analysis.

2×10^5^ cells from homogenized spleens were inoculated into sublethally irradiated recipient mice and the mice were sacrificed at the first appearance of morbidity. Statistics were done using the Mantel-Cox test in GraphPad Prism software (GraphPad Software, La Jolla, CA).

### Analysis of phosphorylated STATs by intracellular flow cytometry

Freshly thawed leukemic and non-leukemic cells (3×10^5^ cells per FACS tube) were fixed with Cytofix buffer (BD Biosciences) according to the manufacturer's protocol followed by permeabilization with cold (−20°C) 90% methanol for 30 min on ice. Cells were incubated with the primary antibody STAT5pY694 Alexa Fluor 647, STAT3pY705 PerCP-Cy5.5, STAT3pS727 Alexa Fluor 488 (BD Biosciences) or IgG control for 40 min at room temperature. After washing with phosphate-buffered saline (PBS) containing 1% FCS and 0.1% sodium azide, cells were measured immediately on a FACS CANTO II (BD Biosciences).

### Cell culture, Apoptosis, ATO treatment, Western Blotting

All cell lines used in this study were purchased from DSMZ. U937 cells were maintained in RPMI 1640 with 10% FCS. For 32D cells the RPMI 1640/10% FCS was supplemented with 10 ng/mL m-IL3. Arsenic trioxide (ATO) (Sigma, Steinheim, Germany) was used at 1μM concentration. Apoptosis was assessed by the 7-amino-actinomycin D (7-AAD) and Annexin V staining method according to the manufacturer's instructions (BD Biosciences) [44].

Western blots were stained with antibodies against RARα, STAT5, JAK2 (Santa Cruz Biotechnology, Santa Cruz, CA, USA), hemagglutinin (α-HA) (Roche, Mannheim, Germany), AML1 (Merck, Darmstadt, Germany), pSTAT3 (Y705), pSTAT5 (Y694), pJAK2 (Y1007/1008) or STAT3 (Cell Signaling, Danvers, MA, USA). For the *in vivo* experiments 5×10^5^ frozen DEK/NUP214-positive leukemic spleen cells were inoculated into sublethally irradiated recipient mice by tail vein injection. Mice were treated with either PBS or 200 mg/day ATO i.p. for 14 days. At first signs of illness the mice were sacrificed and the spleen size was measured.

### Immunohistochemistry

Formalin-fixed paraffin-embedded mouse spleens were cut into 10 μm sections. The tissue slides were deparaffinized, rehydrated in decreasing alcohol concentrations and incubated in 3% H_2_O_2_ for 10 min to block endogenous peroxidase activity. Antigen retrieval was performed at 98°C for 50 min in 10 mM HIER citrate buffer pH 6 (Zytomed Systems, Berlin, Germany) in the water bath. Nonspecific protein binding was blocked with 2% goat serum and 2% BSA in PBS for 30 min. The slides were then incubated with anti-STAT5 antibody (1:200, Santa Cruz Biotechnology) overnight at 4°C. After incubation for one hour with the second anti-rabbit antibody (EnVision+ System-HRP labelled Polymer, DAKO, Hamburg, Germany) the signal was visualized by incubating the slides with AEC substrate (DAKO) and the nuclei were counterstained with hematoxylin (Carl Roth, Karlsruhe, Germany). Spleen sections from mice with FLT3-ITD-driven leukemia or transplanted with mock-transduced HSPCs were used as positive and negative controls, respectively. Staining with the secondary antibody served as further negative control

### Proteomics/Reverse-phase protein array

Proteomic profiling was performed using reverse-phase protein arrays (RPPA) as described previously [34, 35, 45]. Briefly, protein lysates obtained from primary samples were printed onto slides, together with normalization and expression controls, in 5 serial dilutions. The slides were probed with strictly validated primary antibodies against total or phosphorylated protein and a secondary antibody was used to amplify the signal. The stained slides were scanned and the images were analyzed using the MicroVigene Version 3.4 software (VigeneTech Inc. Carlisle, MA, USA) to produce quantified data. The “supercurve” algorithm [46] was used to estimate the sample-specific protein expression levels. The RPPA data were normalized by median centering the results for each sample across all antibodies.

### Statistical analysis

For the statistical analysis (Student's t-test) the Graph Pad Prism software (Graph Pad, San Diego, CA, USA) was used. A p-value of 0.05 was considered statistically significant.

## SUPPLEMENTARY MATERIAL FIGURES


