# Supplementary material for: STAT activation status differentiates leukemogenic from non-leukemogenic stem cells in AML and is suppressed by arsenic in t(6;9)-positive AML
Source: Genes Cancer. 2014 Nov;5(11-12):378–92. doi: 10.18632/genesandcancer.39 (PMC4279436; doi:10.18632/genesandcancer.39)
Supplement: Supplementary file 1 [file ganc-05-378-s001.pdf]

STAT activation status differentiates leukemogenic from non-leukemogenic stem cells in AML and is suppressed by arsenic in t(6;9)-positive AML

Supplementary Material

Immunofluorescence and confocal laser scan analysis of formalin-fixed paraffin embedded (FFPE) tissues sections

Spleen sections of 10 µm were dewaxed in xylene 2 times for 10 min each and rehydrated in graded alcohol series. Epitope retrieval was performed in a water bath at 98°C for 30 min in 10 mM HIER citrate buffer pH 6 (Zytomed Systems, Germany). Unspecific binding was blocked with 5% goat serum and 0.02% Tween20 in TBS for 60 min. Primary antibody STAT5 (Santa Cruz, USA) was diluted 1:200 in blocking solution. The sections were incubated overnight at 4°C followed by extensive washing and incubation for 60 min. at RT with Alexa Fluor® 594 anti-rabbit Ig antibodies (Life Technologies, Darmstadt, Germany). Nuclei were visualized by staining in 0.05 µg/ml 4',6-diamidino-2-phenylindole (DAPI; Sigma). For conventional immunofluorescence staining images were captured on a Axioplan 2 Imaging system (Carl Zeiss, Oberkochen, Germany). For the confocal laser scan analysis images were acquired by a Leica TCS-SP5 system (Leica, Wetzlar, Germany) under identical conditions for pinhole opening, laser power, photomultiplier tension and layer number. During data elaboration by Fiji software (www.fiji.sc) identical parameters were applied for all samples.

Terminal deoxyribonucleotidyl transferase (TdT)-mediated deoxyuridine triphosphate (dUTP) nick end labeling (TUNEL assay)

DNA fragmentation was measured using a TUNEL procedure (DeadEnd™ Colorimetric TUNEL System, Promega, Mannheim, Germany). 10 µm spleen sections were dewaxed in xylene and rehydrated in graded alcohol series to water prior to the staining procedure based on the manufacturer's instructions.

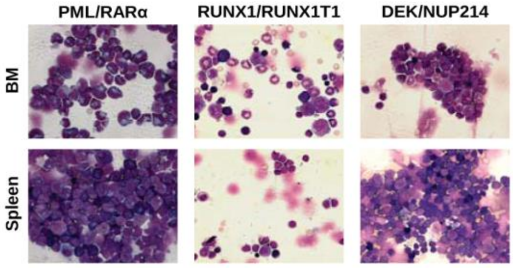

Supplementary Figure S1. Morphology of leukemic blasts. BM and spleen cells from leukemic mice were cytospun and stained with May-Grünwald-Giemsa stain. Here the morphology of blasts from one representative mouse of each group is shown (60x magnification).

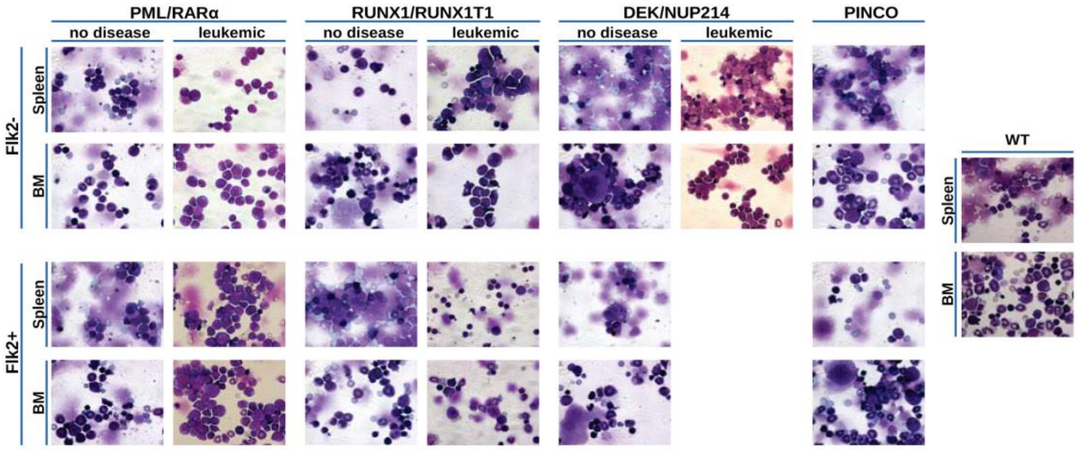

Supplementary Figure S2. **Morphology of BM and spleen cells in leukemic and non-leukemic mice.** The BM and spleen cells of mice inoculated with the Sca1^{+}/c-Kit^{+}/lin^{-}/Flk2^{-} (Flk2-) and Sca1^{+}/c-Kit^{+}/lin^{-}/Flk2^{+} (Flk2+) populations were cytospun and stained with May-Grünwald-Giemsa stain. Cytologic samples from one representative mouse from each group are shown (60x magnification).

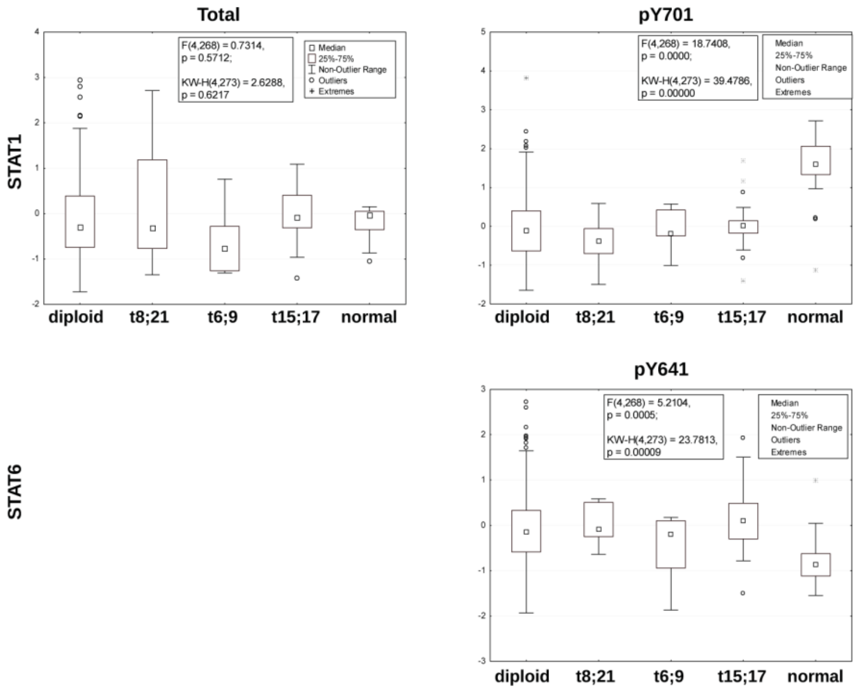

Supplementary Figure S3. **STAT1 and 6 activation in t(6;9), t(15;17) and t(8;21)-positive AML samples**. Box plots showing the expression range of Stat1 (total and pY701) and Stat6 (pY641) stratified by cytogenetic group: Diploid, t(8;21), t(6;9), t(15;17) and normal bone marrow derived CD34+ cells (Normal) are presented. The box shows the 25-75% range with the median shown by the central square (□), outlier by a circle (o) and extremes by an asterisk (*). Statistical results for the F-test and Kruskal-Wallis including the degrees of freedom and the associated p-value are listed.

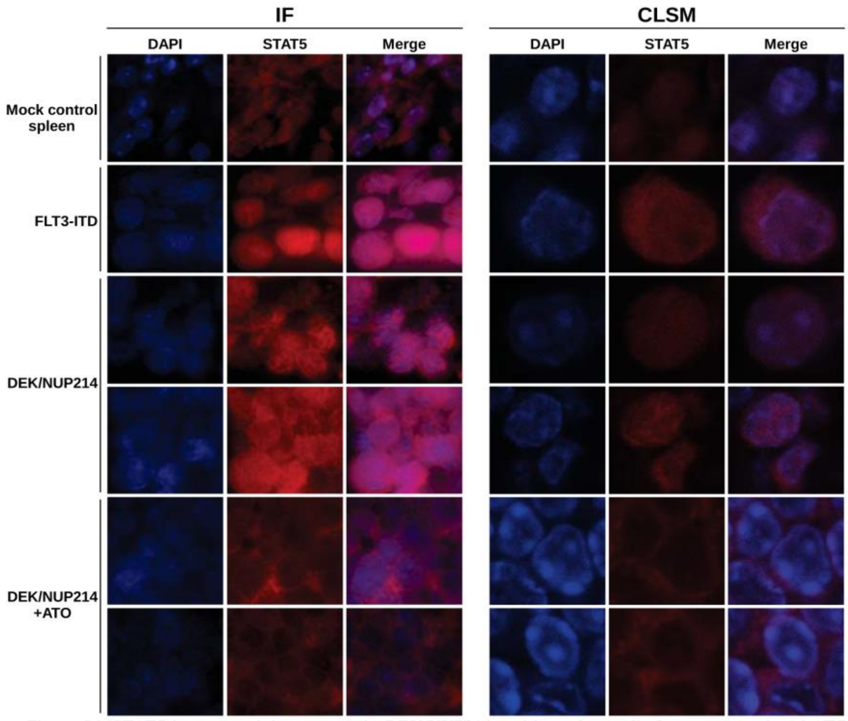

Supplementary Figure S4. **STAT5 immunostaining pattern in DEK/NUP214-positive spleen cells from untreated and ATO-treated mice**. Ten µm sections from paraffin-embedded spleen samples from ATO-treated or untreated mice were stained with rabbit polyclonal anti-STAT5 (red fluorochrome). Nuclei were stained by DAPI (blue staining). IF – immunofluorescence; CLSM – confocal laser scan microscopy. Spleen sections from mice with FLT3-ITD driven leukemia or transplanted with mock-transduced cells served as negative and positive control, respectively. Original magnification 100x.

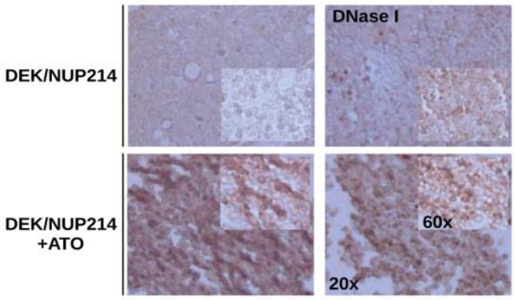

Supplementary Figure S5. **Detection of DNA fragmentation by TUNEL assay as a sign of ongoing apoptosis in DEK/NUP214-positive spleen cells from untreated versus ATO-treated mice**. Ten µm thick sections from paraffin-embedded spleen samples from ATO-treated and untreated mice were processed according to the manufacturer's instructions. One DEK/NUP214-positive spleen section from the untreated group was subjected to DNaseI digestion to generate a positive control for fragmented DNA detection.
